# The *IL1B-511* Polymorphism (rs16944 AA Genotype) Is Increased in Aspirin-Exacerbated Respiratory Disease in Mexican Population

**DOI:** 10.1155/2012/741313

**Published:** 2011-11-03

**Authors:** Ramcés Falfán-Valencia, Gandhi F. Pavón-Romero, Angel Camarena, María de la Luz García, Gustavo Galicia-Negrete, María Cristina Negrete-García, Luis Manuel Teran

**Affiliations:** HLA Laboratory, Department of Immunogenetics and Allergy, Instituto Nacional de Enfermedades Respiratorias, Tlalpan 4502, Sección XVI, Delegación Tlalpan, 14080 México, DF, Mexico

## Abstract

Aspirin exacerbated respiratory disease (AERD) is characterized by chronic hyperplastic rhinosinusitis, nasal polyposis, asthma, and aspirin sensitivity. The mechanisms which produce these manifestations of intolerance are not fully defined, current research focuses on cyclooxygenase 1 (COX-1) inhibition, metabolism of arachidonic acid, and the COX pathway to the lipoxygenase (LO) route, inducing increased synthesis of leukotrienes (LT). The biological plausibility of this model has led to the search for polymorphisms in genes responsible for proinflammatory cytokines synthesis, such as *IL1B* and *IL8*. We performed a genetic association study between *IL8-251* (rs4073) and *IL1B-511* (rs16944) polymorphisms in AERD, aspirin-tolerant asthma (ATA), and healthy control subjects. Using allelic discrimination by real-time PCR, we found statistically nonsignificant associations between AERD, ATA, and healthy control subjects for the GG and GA genotypes of *IL1B* (rs16944). Interestingly, the AA genotype showed an increased frequency in the AERD patients versus the ATA group (GF = 0.19 versus 0.07, *p* = 0.018, OR 2.98, and 95% CI 1.17–7.82). This is the first observation that *IL1B* polymorphisms are involved in AERD. Thus, future studies must investigate whether interleukin-1*β* is released in the airways of AERD patients and whether it relates to genetic polymorphisms in the *IL1B* gene.

## 1. Introduction

Aspirin-exacerbated respiratory disease (AERD) is a syndrome characterized by chronic hyperplastic rhinosinusitis, nasal polyposis, asthma, and aspirin sensitivity, as described in 1922 by SzcZeklik et al. [1, 2]. The prevalence of AERD is variable; Stevenson and Szczeklik reported in 2006 that AERD occurs in 3% of adult patients with asthma in the United States, with the onset of symptoms during the third decade of life, and that it is more common in women than in men, with approximately 70% versus 30% in Europe [2] and 57% versus 43% in the USA [3]. The mechanisms underlying aspirin intolerance are not fully defined, with current research focusing on cyclooxygenase 1 (COX-1) inhibition by aspirin and other NSAIDs diverting arachidonic acid metabolism from COX pathways to the lipoxygenase (LO) pathway. This leads to increased synthesis of the cysteinyl-leukotrienes (LT), LTC_4_, LTD_4_, and LTE_4_, resulting in bronchoconstriction, mucus hypersecretion, and possibly the development of polyps and urticaria [1]. The biological plausibility of this hypothesis fact has led to the search for polymorphisms in genes responsible for LT synthesis, to explore associations between these polymorphisms and local tissue levels of the proteins.

Other factors such as polymorphisms in the genes for proinflammatory cytokines including *TNF*, *IL1B*, *IL6,* and *IL8* are involved in chronic inflammatory and autoimmune diseases. Interleukin 1 (IL-1) is a cytokine associated with inflammatory responses and found in two forms, IL-1*α* (produced by the *IL1A* gene) and IL-1*β* (*IL1B*), with both genes located on chromosome 2. IL-1 is expressed in nasal polyps, nasal epithelium, macrophages, activated T lymphocytes, and monocytes; its expression is regulated by adhesion molecules, and others inflammatory cytokines [4]. *IL1B* polymorphisms have been associated with inflammatory bowel disease and gastric cancer among other diseases [5]. Recently, genetic polymorphisms in proinflammatory cytokines such as IL-1*β* have been recognized as key players in the pathogenesis of asthma [6]. Similarly, IL-8 has been implicated in the asthmatic inflammatory process, and genetic variation in this cytokine has been associated with both the susceptibility and the severity of this disease [7]. In the present study, we have investigated the frequencies of polymorphisms in the genes encoding these two cytokines in AERD patients.

## 2. Materials and Methods

### 2.1. Subjects

Patients with aspirin-exacerbated respiratory disease (AERD) (*n* = 78) and aspirin-tolerant asthma (ATA) (*n* = 135) were recruited from the allergy and otolaryngology departments at the Instituto Nacional de Enfermedades Respiratorias (INER). Healthy control subjects (HCS) (*n* = 134) were invited to participate through AERD-screening campaigns. All participants underwent simple spirometry, inhaled methacholine challenge, and nasal challenge with lysine-aspirin (L-ASA) according to international guidelines to determine the degree of bronchial hyperresponsiveness and confirm AERD diagnosis. The AERD group had positive L-ASA and methacholine challenges, the asthmatic group had positive methacholine challenge, but negative L-ASA challenge, and the healthy control subjects were volunteers with negative L-ASA and methacholine challenges. A positive L-ASA challenge was defined as a decrease of at least 40% in total nasal airflow after L-ASA application compared with baseline measures; methacholine challenge was considered positive with a decrease of at least 20% in forced expiratory volume in one second (FEV_1_) compared with baseline FEV_1_ after the administration of different concentrations of methacholine (beginning with 0.03 mg/mL, increasing gradually the concentration twice every 2 minutes until the concentration of 32 mg/mL); in case it does not have it, it is considered negative. Blood samples were collected for genotyping studies in the HLA laboratory research. The study was approved by the science and bioethics committees of INER, and all participants gave their informed consent [8, 9]. Patients and healthy control subjects had ancestry of at least two generations born in Mexico and were thus considered to be Mexican Mestizo in descent [10, 11].

### 2.2. DNA Extraction

Peripheral blood was drawn by venipuncture, and genomic DNA was obtained using the commercial BDtract DNA isolation kit (Maxim Biotech, San Francisco, Calif, USA). The DNA was quantified by absorption of ultraviolet light at 260 nm wavelength using an ACTGene spectrophotometer (ACTGene, Inc., NJ, USA).

### 2.3. SNP Selection

We selected two polymorphisms in two genes related to chronic inflammation: rs16944 in *IL1B* and rs4073 in *IL8*. Genetic data from each of the polymorphisms are described in Table 1.

### 2.4. Genotyping

Allelic discrimination of SNPs rs16944 (*IL1B*) and rs4073 (*IL8*) was performed by real-time PCR (RT-PCR) on a 7300 Real Time PCR System (Applied Biosystems, Calif, USA) using Taqman commercial probes (Applied Biosystems, USA) for each of the polymorphisms mentioned above and followed the cycling program: preread 50°C, 1 minute; absolute quantitation: 50°C, 2 minutes, 1 cycle; 95°C, 10 minutes, 1 cycle; 95°C, 15 seconds, 60°C 1 min, 40 cycles; postread 50°C, 1 minute. The results were assessed taking into account the allelic discrimination and absolute quantitation in all samples; additionally, we included four contamination controls per plate (nontemplate controls). The interpretation was performed with Sequence Detection Software (v. 1.4). The fluorescence signal detectors used were VIC which was assigned to the B allele and FAM assigned to the A allele for both SNPs.

### 2.5. Statistical Analysis

Statistical analysis was performed between groups of cases (AERD and ATA) versus the healthy control subjects by *χ* ^2^ test with 3 × 2 tables, using SPSS (v. 15.0) software for Windows, to identify the difference between the allele and genotype frequencies of each polymorphism evaluated. A *p*-value <0.05 was considered significant. In addition, odds ratios and 95% confidence intervals were calculated with Epi-info (v. 6.04) software.

## 3. Results

Clinical data for the three groups were compared and are described in Table 2. We performed a genetic association study of the *IL8* (rs4073) and *IL1B* (rs16944) gene polymorphisms in the three groups. Genetic data for the SNPs included in this study are shown in Table 1; minor allele frequency (MAF) of the polymorphisms tested in healthy control subjects had a similar distribution to that reported in international databases (Table 1). Gene frequencies for each genotype within the three subject groups are shown in Table 3. The frequency of genotypes AA, AT, and TT of the *IL8* rs4073 SNP was not statistically significant and different between the three studied groups.

Analysis of GG and GA genotypes of the *IL1B* (rs16944) SNP for AERD and ATA patients versus the healthy control subjects showed nonstatistically significant associations. Interestingly, the AA genotype showed increased frequency in the AERD patients when compared to the ATA group (GF = 0.19 versus 0.07); this association was statistically significant (*p* = 0.018, OR 2.98, and CI 1.17–7.82) (Tables 3 and 4) and was not found when AERD or ATA groups were compared to healthy control subjects. There is no difference in AA versus AG + GG using contrast of healthy control subjects versus AERD patients (data not shown).

## 4. Discussion

The airways of aspirin-sensitive patients are characterized by chronic inflammation with cell infiltration even when they are not exposed to aspirin or other NSAIDs [12]. In addition to alterations in the metabolism of arachidonic acid, several proinflammatory cytokines have been associated with AERD. The role of IL-1, however, has not been investigated previously. Here, we report that AERD patients show an increased frequency of the *IL1B*-511 polymorphism (rs16944 AA genotype) compared to aspirin-tolerant asthmatics.

Interleukin-1*β* has been reported to be involved in the genesis of both asthma [13, 14] and chronic rhinosinusitis with nasal polyposis in a Turkish population [15]. Most studies have attempted to establish the association of polymorphisms in the *IL1B* promoter gene, mainly at positions-511 G/A (rs16944) and -31 C/T. For example, Park et al., in 2004, did not find any association between these polymorphisms and either asthma or atopy in a Korean population [16]. In 2007, Erbek et al. described a susceptibility for developing nasal polyps associated with the *IL1B-511* polymorphism (rs16944) [15], but Mfuna Endam et al., in 2010, failed to reproduce this finding in Canadian patients with chronic rhinosinusitis [17]. In 2003, Allen et al. did not find any association with *IL1* gene polymorphisms in asthmatic families (*n* = 244), but they reported an association with the DNA microsatellite D2S308 in these asthma families (*p *= 0.00001) [18]. In parallel, Karajalein et al. evaluated 245 patients with asthma and nasal polyposis and did not find any association between the polymorphism *IL1B-511* C/T and nasal polyps [19]. Evidence for the role of IL-1 in pulmonary immune responses has been gathered in murine models of allergic asthma using IL-1R1-deficient [IL-1R1 (−/−)] mice; changes observed in these mice include significant reduction of pulmonary eosinophilic inflammation, diminished goblet cell hyperplasia, and reduction of cell recruitment to the lungs, as compared to control BALB/c mice [20]. However, there are no studies linking gene promoter polymorphisms and levels of expression of the cytokine in the lung microenvironment.

In the present study, we have demonstrated that the frequency of the *IL1B-511* polymorphism (rs16944 AA genotype) is three-fold higher in AERD (19.2%) than in ATA patients (7.4%), suggesting that patients carrying this polymorphism may exhibit genetic susceptibility to develop AERD.

Findings on the biological functionality of the rs16944 polymorphism have not been consistent across studies. The AA genotype has been associated with higher gastric mucosal levels of IL-1*β* in bacterial infections [21], while mononuclear cells from subjects with the GG genotype showed an increased release of IL-1*β* after stimulation with lipopolysaccharide [22]. Recent studies suggest that the functional role of rs16944 may depend on *IL1B* promoter region haplotypes including rs16944 [23–26]. Although the findings are inconsistent, these previous studies suggest that rs16944 could affect the expression levels of IL-1*β*. Our report is the first demonstration of the involvement of *IL1B* polymorphism in AERD. The sample size is relatively small, particularly in the AERD group, which may limit the statistical power, so it would be desirable to replicate our findings in an independent population.

Future studies must investigate whether this cytokine is released in the airways of AERD patients and whether its levels relate to genetic polymorphisms in the *IL1B* gene.

Interleukin-8 has been implicated in asthma and found in high concentrations in bronchoalveolar lavage fluid of patients with acute asthma exacerbations [26]. In fact, polymorphic *IL8* alleles (−251T and 781C) have been associated with asthma in a European population [7, 27], but not in asthmatics of Korean origin [7]; the differences could be explained by the different ethnic populations studied. In contrast, Korean asthmatics were found to show four nonsynonymous amino acid substitutions in the IL-8 receptor A (IL8RA) and an association of one synonymous variation in IL8RB [28]. In the present study, we did not find a significant difference in the rs4073 A/T between the AERD group and ATA patients.

## Figures and Tables

**Table 1:** Genetic data on SNPs investigated in study.

SNP	Chr	Gene		Alleles		
		Symbol	Position	Change	Ancestral	MAF
rs4073	4	*IL8*	−251	A/T	A	*T* = 0.492
rs16944	2	*IL1B*	−511	G/A	A	*A* = 0.462

Chr: Chromosome, MAF: Minor allele frequency, MAF source: 1000 genomes phase 1 from dbSNP.

**Table 2:** Summary of clinical characteristics of AERD, ATA, and HCS.

	AERD	ATA	HCS
Subjects	78	135	134
Gender (male/female)	31/47	49/86	79/55
Mean age (years, SD)	42.0 (14.4)	36.7 (17)	24 (8.9)
Premethacholine challenge FEV_1_ (%)	99.6 (17.3)	101.4 (12.9)	98.2 (13.3)
Postmethacholine challenge FEV_1_ (%)	75.1 (13.8)	76.9 (14.4)	95.4 (11.6)
Pre-L-ASA challenge nasal flow (mL/sec)	640.75 (162.2)	689 (178.2)	670.2 (167.5)
Post-L-ASA challenge nasal flow (mL/sec)	488 (142.8)	658.5 (186.3)	682.4 (165.7)

AERD: Aspirin-exacerbated respiratory disease, ATA: Aspirin-tolerant asthma, HCS: Healthy control subjects, SD: Standard deviation, FEV_1_: Forced expiratory volume in one second, L-ASA: Lysine aspirin.

**Table 3:** Genotype frequencies of *IL8* and *IL1B* genes in AERD, ATA, and HCS.

Gene/SNP Genotype	AERD		ATA		HCS	
	*n* = 78	GF (%)	*n* = 135	GF (%)	*n* = 134	GF (%)
*IL8*						
rs4073						
AA	35	0.449 (44.87)	54	0.400 (40.00)	53	0.396 (39.55)
AT	33	0.423 (42.31)	57	0.422 (42.22)	63	0.470 (47.01)
TT	10	0.128 (12.82)	24	0.178 (17.78)	18	0.134 (13.43)
*IL1B*						
rs16944						
GG	23	0.295 (29.49)	57	0.422 (42.22)	47	0.351 (35.07)
GA	40	0.513 (51.28)	68	0.504 (50.37)	72	0.537 (53.73)
AA	15	0.192 (19.23)	10	0.074 (7.41)	15	0.112 (11.19)

AERD: Aspirin-exacerbated respiratory disease, ATA: aspirin-tolerant asthma, HCS: healthy control subjects, GF: genotype frequency.

**Table 4:** Statistical association of *IL1B* rs16944 AA in AERD and ATA patients.

Gen/SNP	AERD		ATA		*P*	OR	CI (95%)
	*n* = 78	GF (%)	*n* = 135	GF (%)			
*IL1B*/rs16944							
AA	15	0.192 (19.23)	10	0.074 (7.41)	0.018	2.98	1.17–7.82

AERD: Aspirin-exacerbated respiratory disease, ATA: aspirin-tolerant asthma, GF: genotype frequency, *p* value Yates correction's, OR: odds ratio, 95% IC: 95% Confidences Interval.

## References

[1] Stevenson DD, Szczeklik A (2006). Clinical and pathologic perspectives on aspirin sensitivity and asthma. *Journal of Allergy and Clinical Immunology*.

[2] Szczeklik A, Nizankowska E, Duplaga M (2000). Natural history of aspirin-induced asthma. *European Respiratory Journal*.

[3] Berges-Gimeno MP, Simon RA, Stevenson DD (2002). The natural history and clinical characteristics of aspirin-exacerbated respiratory disease. *Annals of Allergy, Asthma and Immunology*.

[4] Otto BA, Wenzel SE (2008). The role of cytokines in chronic rhinosinusitis with nasal polyps. *Current Opinion in Otolaryngology and Head and Neck Surgery*.

[5] Machado JC, Pharoah P, Sousa S (2001). Interleukin 1B and interleukin 1RN polymorphisms are associated with increased risk of gastric carcinoma. *Gastroenterology*.

[6] Chu JH, Weiss ST, Carey VJ, Raby BA (2009). A graphical model approach for inferring large-scale networks integrating gene expression and genetic polymorphism. *BMC Systems Biology*.

[7] Heinzmann A, Ahlert I, Kurz T, Berner R, Deichmann KA (2004). Association study suggests opposite effects of polymorphisms within IL8 on bronchial asthma and respiratory syncytial virus bronchiolitis. *Journal of Allergy and Clinical Immunology*.

[8] Cockcroft DW (2008). Methacholine challenge methods. *Chest*.

[9] Nizankowska-Mogilnicka E, Bochenek G, Mastalerz L (2007). EAACI/GA2LEN guideline: aspirin provocation tests for diagnosis of aspirin hypersensitivity. *Allergy*.

[10] Lisker R, Perez-Briceno R, Granados J, Babinsky V (1988). Gene frequencies and admixture estimates in the State of Puebla, Mexico. *American Journal of Physical Anthropology*.

[11] Lisker R, Ramirez E, Briceno RP, Granados J, Babinsky V (1990). Gene frequencies and admixture estimates in four Mexican urban centers. *Human Biology*.

[12] Stevenson DD, Zuraw BL (2003). Pathogenesis of aspirin-exacerbated respiratory disease. *Clinical Reviews in Allergy and Immunology*.

[13] Karjalainen J, Nieminen MM, Aromaa A, Klaukka T, Hurme M (2002). The IL-1*β* genotype carries asthma susceptibility only in men. *Journal of Allergy and Clinical Immunology*.

[14] Karjalainen J, Hulkkonen J, Pessi T (2002). The IL1A genotype associates with atopy in nonasthmatic adults. *Journal of Allergy and Clinical Immunology*.

[15] Erbek SS, Yurtcu E, Erbek S, Atac FB, Sahin FI, Cakmak O (2007). Proinflammatory cytokine single nucleotide polymorphisms in nasal polyposis. *Archives of Otolaryngology—Head and Neck Surgery*.

[16] Park BL, Kim LH, Choi YH (2004). Interleukin 3 (IL3) polymorphisms associated with decreased risk of asthma and atopy. *Journal of Human Genetics*.

[17] Mfuna Endam L, Cormier C, Bossé Y, Filali-Mouhim A, Desrosiers M (2010). Association of IL1A, IL1B, and TNF gene polymorphisms with chronic rhinosinusitis with and without nasal polyposis: a replication study. *Archives of Otolaryngology—Head and Neck Surgery*.

[18] Allen M, Heinzmann A, Noguchi E (2003). Positional cloning of a novel gene influencing asthma from Chromosome 2q14. *Nature Genetics*.

[19] Karjalainen J, Joki-Erkkilä VP, Hulkkonen J (2003). The IL1A genotype is associated with nasal polyposis in asthmatic adults. *Allergy*.

[20] Schmitz N, Kurrer M, Kopf M (2003). The IL-1 receptor 1 is critical for Th2 cell type airway immune responses in a mild but not in a more severe asthma model. *European Journal of Immunology*.

[21] Hwang IR, Kodama T, Kikuchi S (2002). Effect of interleukin 1 polymorphisms on gastric mucosal interleukin 1*β* production in Helicobacter pylori infection. *Gastroenterology*.

[22] Iacoviello L, Di Castelnuovo A, Gattone M (2005). Polymorphisms of the interleukin-1*β* gene affect the risk of myocardial infarction and ischemic stroke at young age and the response of mononuclear cells to stimulation in vitro. *Arteriosclerosis, Thrombosis, and Vascular Biology*.

[23] Chen H, Wilkins LM, Aziz N (2006). Single nucleotide polymorphisms in the human interleukin-1B gene affect transcription according to haplotype context. *Human Molecular Genetics*.

[24] Wen AQ, Wang J, Feng K, Zhu PF, Wang ZG, Jiang JX (2006). Effects of haplotypes in the interleukin 1*β* promoter on lipopolysaccharide-induced interleukin 1*β* expression. *Shock*.

[25] Wen AQ, Gu W, Wang J (2010). Clinical relevance of IL-1*β* promoter polymorphisms (-1470,-511, and-31) in patients with major trauma. *Shock*.

[26] Hall SK, Perregaux DG, Gabel CA (2004). Correlation of polymorphic variation in the promoter region of the interleukin-1*β* gene with secretion of interleukin-1*β* protein. *Arthritis and Rheumatism*.

[27] Puthothu B, Krueger M, Heinze J, Forster J, Heinzmann A (2006). Impact of IL8 and IL8-Receptor alpha polymorphisms on the genetics of bronchial asthma and severe RSV infections. *Clinical and Molecular Allergy*.

[28] Cheong HS, Shin HD, Lee SO (2006). Polymorphisms in interleukin 8 and its receptors (IL8, IL8RA and IL8RB) and association of common IL8 receptor variants with peripheral blood eosinophil counts. *Journal of Human Genetics*.

